# Redefining Board Certified Toxicologist by Consumer Products Safety Commission May Increase Potential Risk of Exposure to Carcinogens among Consumers in United States of America

**DOI:** 10.3389/fpubh.2017.00029

**Published:** 2017-02-28

**Authors:** James L. Unmack, Vergil C. Bud Jenkins, Masood A. Shammas

**Affiliations:** ^1^Unmack Corporation, San Pedro, CA, USA; ^2^Coatings Scientist Consulting Services, Riverside, CA, USA; ^3^Department of Adult Oncology, Harvard (Dana Farber) Cancer Institute, Boston, MA, USA

**Keywords:** toxicological review, art material, carcinogenesis, toxicologist, Consumer Products Safety Commission

Toxicological Review of art material formulations in the United States of America is required to be done by a toxicologist certified by a nationally recognized board in Toxicology (Federal Hazardous Substance Act 16CFR1500). The only two boards listed on the Consumer Products Safety Commission (CPSC) web site meeting this qualification are the American Board of Toxicology (ABT) and The Academy of Toxicological Sciences. This definition seems to have been changed by CPSC toxicologist. We feel that this is an error in judgment, which is not compatible with the mission of ABT and could possibly harm American consumers. We want to bring attention to this fact so it can be changed back to the *status quo* ante.

The use of art materials in the United States of America is very common. In general, the use of art materials by the United States of America’ public starts at a very early age and continues through adolescence onto senior status and most probably up to the time a person reaches retirement or in a rehabilitation home. In the United States of America, art materials are not only used by professionals but also used by a huge number of small children in elementary schools as well as adults in colleges, universities, and homes. According to the survey conducted by the National Art Materials Association (NAMTA) in year 2015, 22 million adults in the United States of America created paintings, drawings, or sculptures. Moreover, the trend of using art materials in the United States of America appears to be on the rise. According to the survey conducted by NAMTA, the art supplies industry grew about 4% per year between 2012 and 2014 with about 640 specialty art material stores in the United States of America with a total of 1.5 billion dollar sales during the year. It is known that art materials may contain chemicals, which are associated with chronic toxicity (1, 2). Some of these chemicals include heavy metals such as nickel chloride can potentially dysregulate mechanisms involved in genome maintenance and repair (3, 4) and may predispose human cells to oncogenesis. We have shown that homologous recombination (HR), a major DNA repair mechanism, is overactive and dysregulated in Barrett’s adenocarcinoma (5) and multiple myeloma cells (4). Elevated HR in these cells serves as a key mechanism in the acquisition of new genomic changes over time and significantly contributes to development of resistance to treatment (4) and growth of tumor cells in animal models (6). We also show that exposure of human cancer cells to heavy metals such as nickel (4) further increases HR activity in these cells. These data suggest that products containing chemicals such as heavy metals could have considerable health risk, especially if used—over a long period of time. Moreover, if more than one heavy meta and/or other toxic chemicals are found in a product, their harmful effects could be combined with each other and/or other intracelluar factors leading to significantly increased risk. Consistent with this view point, it has been demonstrated that toxic effects of heavy metals are substantially increased when combined with each other or when X-rays (1, 2) are used. We, therefore, emphasize that art materials, especially if used over a long period of time, have a considerable health risk and to be on safe side, their safety and compliance must be established by highly qualified US Certified Toxicologists, such as DABTs.

To minimize the hazard from chronic toxicity, the Labeling of Hazardous Art Materials Act (LHAMA) (7), 15 U.S.C 1277 (Public L. 100-695) was enacted on November 18, 1988. The Labeling of Hazardous Art Materials Act requires that art material sold in the United States of America be toxicologically reviewed by qualified toxicologists for any potential adverse chronic health effects on consumers, and to ensure proper labeling of chronic hazards (4). Under the Federal Hazardous Substance Act Regulation at 16 CFR 1500.14(b)(8) (7), the qualified toxicologist is required to be Board Certified by a Nationally Recognized Certification Board. The regulation states that a qualified toxicologist is an individual who through education, training, and experience has expertise in the field of toxicology, as it relates to human exposure and is either a toxicologist or physician certified by a nationally recognized board such as the ABT.

The vision of the ABT is to establish a globally recognized credential in toxicology, which is representative of competency and commitment to human health and environmental sciences and to identify, maintain, and evolve a standard for professional competency in the field of toxicology (8). Thus, the very purpose of ABT is to ensure that a toxicologist has proper education and experience. However, few years ago, the CPSC (9) re-defined a Board Certified Toxicologist (DABT) for the purpose of LHAMA. According to new definition (1) a toxicologist is one who has knowledge, experience, and education in assessing risk; and (2) an experienced individual is not required to have Board Certification. This new definition by the CPSC not only questions the importance of the role of ABT but also gives an impression as if CPSC has dropped its standard for ensuring art materials safety, especially those being imported from other countries.

Moreover, ABT requires recertification of their diplomats after every 5 years. The purpose of the recertification program is to make sure that the diplomats remain up-to-date with continuing developments in the field of toxicology, especially because this is a rapidly developing science. Without this program, excellence in the profession of toxicology cannot be expected. To improve the quality of their diplomats, the ABT has set the following three performance criteria against, which diplomats are evaluated during recertification process: (1) active practice of toxicology; (2) continuing education; (3) and maintaining expert knowledge in toxicology. Since new information in science is added every day, continuing education of a toxicologist and continuing verification of his/her credentials are extremely important to ensure the accuracy and validity of a toxicological review. For example, biological research in recent years has identified an important role of tumor microenvironment (i.e., cells and factors surrounding tumor including fibroblasts, stromal cells, endothelial cell precursors, lymphocytes, antigen-presenting cells, innate immune cells, and cytokines) in survival, growth and/or progression of tumor cells (10, 11). Therefore, disruption/alteration of tumor microenvironment by a chemical can be another potential mechanism of carcinogenesis (12). This suggests that a chemical affecting physical interaction and/or crosstalk among various components of microenvironment could be a potential carcinogen. Similarly, e-cigarettes, once considered to be a safer alternative, have recently been shown to be associated with heavy metal exposure (13). Moreover, new substances with potential to cause health risk are identified all the time. For example, Table 1 provides few examples of the new chemicals that were added last year to the list of agents known to the State of California (USA) to cause developmental toxicity and/or cancer. This shows how rapidly the science is evolving. It is, therefore, extremely important that a toxicologist maintains his/her expert knowledge through continuing education.

**Table 1:** **Examples of new chemicals added by The California’s Office of Environmental Health Hazard Assessment to the list of chemicals known to the state to cause developmental toxicity and/or cancer, for purposes of the Safe Drinking Water and Toxic Enforcement Act of 1986 (Proposition 65)**.

	Name of chemical	Date the chemical was added to list of harmful chemicals	Risk
1	Abiraterone acetate (CAS# 154229-18-2)	April 8, 2016	Developmental toxicity both in males and females
2	Atrazine (CAS# 1912-24-9)	July 15, 2016	Developmental toxicity in females
3	Bromodichloroacetic acid (CAS# 71133-14-7)	July 29, 2016	Cancer
4	1-Bromopropane (1-BP) (CAS# 106-94-5)	August 5, 2016	Cancer and developmental toxicity in females
5	1-Des-ethyl atrazine (DEA) (CAS# 6190-65-4)	July 15, 2016	Developmental toxicity in females
6	1-Des-isopropyl atrazine (DIA) (CAS# 1007-28-9)	July 15, 2016	Developmental toxicity in females
7	1-2,4-Diamino-6-chloro-s-triazine (DACT) (CAS# 3397-62-4)	July 15, 2016	Developmental toxicity in females
8	Malathion (CAS# 121-75-5)	May 20, 2016	Cancer
9	Sedaxane (CAS# 874967-67-6)	July 1, 2016	Cancer
10	Simazine (CAS# 122-34-9)	July 15, 2016	Developmental toxicity to females

*To limit the number of examples, we have only listed part of the changes which took place in the year 2016 and are related to carcinogens and reproductive toxins*.

The problem is that the new definition may allow at least some unqualified or under qualified individuals to conduct toxicological reviews for art materials to be imported into the United States of America. This is because now there is no way to verify credentials and the current status of the knowledge of individuals who conduct toxicological reviews of art material formulations for proper labeling. As a result, anyone can claim to be a qualified toxicologist for the purpose of LHAMA. This is expected to happen more often in countries where laws are either soft or are not followed properly as are being done in United States of America. Unfortunately, these are the countries where most of the art materials are manufactured for export to the United States of America.

In our opinion, the CPSC’s shift in definition may not be in the best interest of consumers, especially children, as it relates to exposure to chronic toxicants, including carcinogens and reproductive toxins. We, therefore, suggest that CPSC should consider reversing its decision about redefining “Board Certified Toxicologist” for the purpose of LHAMA and leave the way it was in the original regulation. This will ensure the maintenance of a high standard of art material safety and protect the welfare of consumers.

## Author Contributions

JU identified the problem and assisted in manuscript preparation; VJ critically evaluated the problem, provided relevant details, and assisted in organization and preparation of manuscript; MS supervised the project, explained the relevance to human health, and prepared the manuscript.

## Conflict of Interest Statement

The authors declare that the research was conducted in the absence of any commercial or financial relationships that could be construed as a potential conflict of interest.
